# Genetic Inhibition of Plppr5 Aggravates Hypoxic-Ischemie-Induced Cortical Damage and Excitotoxic Phenotype

**DOI:** 10.3389/fnins.2022.751489

**Published:** 2022-03-24

**Authors:** Yuxiao Sun, Mei-fang Jin, Lili Li, Yueying Liu, Dandan Wang, Hong Ni

**Affiliations:** ^1^Division of Brain Science, Institute of Pediatric Research, Children’s Hospital of Soochow University, Suzhou, China; ^2^The First Affiliated Hospital of Zhejiang University School of Medicine, Hangzhou, China; ^3^Department of Pediatrics, Affiliated Hospital of Jiangnan University, Wuxi, China

**Keywords:** plppr5, knockout, excitotoxic, hypoxic-ischemia, neonatal

## Abstract

Hypoxia-ischemia (HI) is the most common acute brain threat in neonates and a leading cause of neurodevelopmental impairment. Exploring the new molecular mechanism of HI brain injury has important clinical translational significance for the next clinical intervention research. Lipid phosphatase-related proteins (PLPPRs) are regulators of mitochondrial membrane integrity and energy metabolism. We recently found that *Plppr5* knockout exacerbated HI impairment in some aspects and partially attenuated the neuroprotective effects of melatonin, suggesting that *Plppr5* may be a novel intervention target for HI. The present study aimed to determine the long-term effects of gene knockout of *Plppr5* on HI brain injury, focusing on the neuronal excitability phenotype, and to determine the effect of *Plppr5* gene silencing on neuronal zinc metabolism and mitochondrial function *in vitro*. 10-day-old wild type (WT) mice and *Plppr5*-deficient (*Plppr5*^–/–^) mice were subjected to hypoxia-ischemia. Lesion volumes and HI-induced neuroexcitotoxic phenotypes were quantified together with ZnT1 protein expression in hippocampus. In addition, HT22 (mouse hippocampal neuronal cells) cell model was established by oxygen–glucose deprivation/reoxygenation (OGD/R) treatment and was treated with medium containing LV-sh_*Plppr5* or control virus. Mitochondrial oxidative stress indicator ROS, mitochondrial ZnT1 protein expression and zinc ion content were detected.

**Results:**

*Plppr5*-deficient mice subjected to hypoxia-ischemia at postnatal day 10 present significantly higher cerebral infarction. *Plppr5*-deficient mice were endowed with a more pronounced superexcitability phenotype at 4 weeks after HI, manifested as a reduced seizure threshold. ZnT1 protein was also found reduced in *Plppr5*-deficient mice as well as in mice subjected to HI excitotoxicity. *Plppr5* knockout *in vivo* exacerbates HI brain injury phenotypes, including infarct volume and seizure threshold. In addition, knockout of the *Plppr5* gene reduced the MFS score to some extent. *In vitro Plppr5* silencing directly interferes with neuronal zinc metabolism homeostasis and exacerbates hypoxia-induced mitochondrial oxidative stress damage. Taken together, our findings demonstrate for the first time that *Plppr5*-deficient mouse pups exposed to neuronal hypoxia and ischemia exhibit aggravated acute brain injury and long-term brain excitability compared with the same treated WT pups, which may be related to the disruption of zinc and mitochondria-dependent metabolic pathways in the hippocampus. These data support further investigation into novel approaches targeting *Plppr5*-mediated zinc and mitochondrial homeostasis in neonatal HIE.

## Introduction

Neonatal Hypoxic-Ischemic Brain Damage (HIBD) refers to neonatal brain damage caused by cerebral hypoxia and ischemia, mostly caused by neonatal asphyxia that occurs in the perinatal period. The incidence of HIBD in live births is about 3‰∼6‰, of which about 18% die in the neonatal period, and about 25% of surviving children also have permanent neurological damage (Horn et al., 2013). Every year, more than 2 million babies die or develop permanent sequelae due to HIBD in the world (Martínez-Orgado et al., 2020). A report by The Lancet showed that about 75% of children with HIBD had seizures, and some children developed epilepsy (Gluckman et al., 2005). At present, several types of drugs commonly used in clinical treatment of convulsions caused by HIBD have many adverse consequences (Mitra et al., 2016). The HIBD animal model will help to reveal the mechanism of HIBD-induced epileptogenesis and provide new clues for early intervention.

The molecular and biochemical mechanisms of acute neonatal hypoxic-ischemic brain injury mainly include intracellular free radical/ion-mediated cell death/survival gene expression and mitochondrial dysfunction. However, the long-term effects of hypoxia-ischemia on brain function, especially on brain excitability and epileptogenesis, are still poorly investigated. Epileptogenesis refers to the pathophysiological process from the initial stimulation of brain injury to the final formation of spontaneous epilepsy (Pitkänen and Lukasiuk, 2009). The classic neuroplasticity change during the incubation period after the initial brain injury is the abnormal distribution and enhancement of active zinc ion staining (Timm staining) in hippocampal mossy fibers (MFs) (regenerative sprouting) (Rakhade and Jensen, 2009). The brain is rich in zinc, which is an important trace metal element in the central nervous network system. The normal survival and development of cells depend on zinc homeostasis. Zinc has two sides to brain mitochondrial function. Physiological concentration of zinc might be beneficial in protecting mitochondrial antioxidants from oxidative stress damage (Adebayo et al., 2016). However, glutamate-induced zinc release and dyshomeostasis contributed to HT22 neuronal mitochondrial injury (Jin et al., 2018).

Plasticity-Related Gene (also known as Phospholipid Phosphatase Related, PLPPR) is a new member of the brain-specific lipid phosphatase superfamily, including plasticity-related gene family 1-5 (PLPPR1-5) (Peeva et al., 2006; Sigal et al., 2007; Bräuer and Nitsch, 2008; Tang and Brindley, 2020). Our previous study demonstrated long-term abnormal expression of hippocampal zinc ion transporter 1 (ZnT-1), zinc ion transporter 3 (ZnT3), PLPPR4, and PLPPR1 induced by developmental seizures, and the high correlation among these molecules is also destroyed (Ni et al., 2009a,b), indicating that both PLPPR and zinc ion transporter signals are involved in the regulation of long-term hippocampal MF sprouting following neonatal seizures. However, the effect of *Plppr5* on neonatal hypoxic-ischemic brain damage and its relationship with zinc ion signal remains unclear.

The aim of this study was to compare *Plppr5*^–/–^ mice and their littermate wild-type mice at 4 weeks after HIBD treatment. The main observation parameters include the seizure threshold, the distribution and intensity of active zinc ion staining (Timm staining, sprouting) of hippocampal mossy fibers, and the level of ZnT-1 protein expression. We also further revealed the internal connection between *Plppr5*, mitochondrial damage and zinc ion signal through *in vitro* cell experiments, providing new ideas for the clinical treatment of HIBD.

## Materials and Methods

### Experimental Animals

**FIGURE 1:**
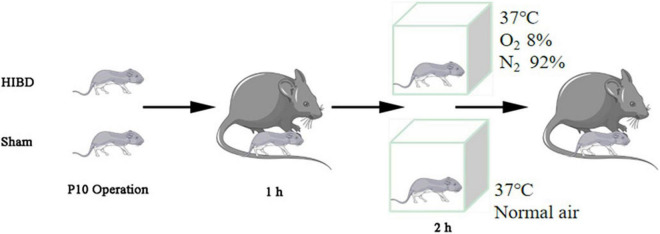
HIBD model making.

The generation of *Plppr5* knockout mice was conducted in the GemPharmatech Co., Ltd. (Nanjing, China) using CRISPR/Cas9 technology as has been reported before (Sun et al., 2021). KO and littermate wildtype (WT) mice of 10 days were used in our experiments and only male mice were used. All experiments were conducted with approval of the University of Soochow University Animal Care and Use Committee (ethical code:SUDA20201020A01). Mice were tested blindly for genotype. Hypoxic-Ischemic Brain Damage (HIBD) is based on the mature Rice Vannucci model (Rice et al., 1981). Briefly, 10-day old mice were anesthetized, then, the left common carotid artery was carefully separated and cut in the middle after double ligation with a 5–0 suture. Lastly, the skin was sutured. The entire procedure took no more than 6 min. The mice were returned to the dam to recover for one hour and then placed in a hypoxic chamber for two hours (37°C, 8% oxygen, 92% nitrogen). The sham-operated (Sham) mice were only treated by isolating the left common carotid artery without ligation, and placed in a similar equipment without hypoxic (Figure 1). A total of thirty male WT mice and thirty *Plppr5*^–/–^ mice were randomly selected and divided into the following four groups (fifteen in each group): WT group (wild-type sham operation group, *n* = 15), KO group (*Plppr5*^–/–^ mice with sham operation group, *n* = 15), WT + HIBD group (wild-type mice with hypoxic-ischemic group, *n* = 15), KO + HIBD group (*Plppr5*^–/–^mice with hypoxic-ischemic group, *n* = 15).

### Measurement of Infarct Volume

Three mice in each group were anesthetized and executed 24 h after HIBD. The brains were removed and sliced coronally into 2-mm-thick sections and incubated in 1% 2,3,5-triphenyltetrazolium chloride (TTC) (Sigma, St. Louis, MO, United States) at 37°C in the dark for 10 min. Then fixed overnight in 4% formaldehyde solution. The TTC-stained brain sections were photographed and the infarct volumes were measured by Image J (an image analysis software, United States). The degree of cerebral infarction is presented as the percentage of infarction volume to total brain volume (Wang et al., 2016; Wong et al., 2018; Omur et al., 2019).

### Determination of Seizure Threshold

To explore the difference of the seizure latency (seizure threshold) between hypoxic-ischemic brain injury and sham operation, and the effect of *Plppr5* knockout.

Four weeks after HIBD, six mice in each group were randomly selected to test. The specific operation refers to the literature (Jin et al., 2018): the mice were injected with penicillin (5.1 × 10^6^U/kg/d, i.p.), and the time from the injection of penicillin to the convulsive seizure of the mouse were recorded. The observation time was 90 min. The intensity of epilepsy was determined according to the Racine classification (Racine, 1972). Briefly, stage 1, stereotype mouth movement, eye blinking and/or mild facial clonus; stage 2, head nodding and/or sever facial clonus; stage 3, unilateral forelimb clonic twitching, but no hindlimb erection; stage 4, clonic convulsions in the forelimbs with rearing; and stage 5, generalized tonic-clonic seizures with falls, loss of postural control. Moreover, after the seizure started, the mouse was injected with 4% chloral hydrate immediately.

### Neo-Timm’s Staining

The neo-Timm’s staining is based on the sulfide/silver method. Three mice were randomly selected from each group four weeks after HIBD. After anesthesia, they were fixed in the supine position. The chest was opened along the midline and the heart was exposed. Then, PBS, 0.4% Na_2_S, 4% paraformaldehyde, 0.4% Na_2_S were injected into the heart in turn. Then removed the brain and put into 4% paraformaldehyde for fixation, and selected according to the hippocampal positioning of Wiqas et al. (2020). The section with the largest area of the dorsal hippocampus (Bregma-1.3 to -3.7 mm, The mouse brain in stereotaxic coordinates. Amsterdam, the Netherlands, Boston, MA: Elsevier/Academic Press.). Incubate the Timm staining solution (90 ml of 50% gum Arabic with 7.65 g of citric acid, 7.05 g of sodium citrate in 22 ml ddH_2_0, 1.5 ml of 17% AgNO_3_, 5.3 g of hydroquinone in 90 ml ddH_2_O) at 37°C for 50 min in the dark. After the incubation, rinse it in running water and pass the 70%, 80%, 90% and 100% staining solution one by one. It is dehydrated in ethanol solution and mounted with neutral resin (Hsu and Buzsáki, 1993; Ni et al., 2009b; Yang et al., 2013). After drying, observe and take pictures under a light microscope. Semi-quantitative scoring of mossy fiber sprouting (MFS) in hippocampal DG and CA3 regions was performed (the scoring was done by persons who did not know the experimental group), and 5 consecutive brain slices were observed for each brain tissue CA3 area and DG area (Holmes et al., 1999; Ni et al., 2009b).

### Cell Culture

HT22 cells (neuronal line of mouse hippocampus) were obtained from the Cell Bank of the Institute of Cell Biology, Chinese Academy of Sciences. Cells were cultured in medium composed of DMEM, 10% FBS, 100U/mL penicillin, and 100mg/mL streptomycin in the cell culture incubator at 37°C with 5% CO_2_.

### Viral Infecton and Oxygen Glucose Deprivation/Reoxygenation (OGD/R) of HT22 Cells

For viral infection *in vitro*, when cells grew to 60–70% confluency, the HT22 cells were transfected with medium containing LV-*Plppr5*-RNAi (MOI = 100), and empty plasmids LV-control-RNAi (normal control;MOI = 100; Genechem). After 24 hours of transfection, serum-free transfer solution was replaced by complete medium to culture for 48 h. The cells were cultured for 3 days and then subjected to OGD/R. Oxygen and glucose deprivation/reoxygenation (OGD/R) is an accepted *in vitro* model for simulating HIBD. First, HT22 cells were cultured in glucose-free DMEM in a hypoxia incubator chamber (Billups-Rothenberg, Inc.) with 1% O_2_, 5% CO_2_, and 37°C for 6 h. Then, the medium was replaced with complete DMEM and the cells were incubated in an incubator with 5% CO_2_ at 37°C for another 4h to simulated the reperfusion. Cells in the control group were treated identically except that they were not exposed to OGD (Wei et al., 2017). Briefly, it was divided into four groups: Sh-control, Sh-*Plppr5*, Sh-control + OGD/R, Sh-*Plppr5* + OGD/R.

### Western Blot

Brain tissues from the left hippocampus region of the mice or cells were collected and added with RIPA lysis buffer (Beyotime Biotechnology, China) (Rafało-Ulińska et al., 2020). Lyse on ice for 1 h after shaking. The supernatant was collected after centrifugation at 4°C and 12,000 × g for 30 min. Protein concentrations were determined with BCA protein assay kit (Beyotime Biotechnology, China). Next, the protein extracts were boiled at 100°C for 10 min. Equal amount of protein from each sample was resolved on SDS–PAGE, trans-blotted onto polyvinylidene fluoride membranes (Millipore), and subjected to immunoblot assay by primary antibodies followed by secondary antibodies. The following antibodies were used for western blotting: rabbit polyclonal anti-ZnT1 (SLC30A1) antibody (1:1000; allomone labs, AZT-011), and mouse monoclonal beta actin antibody (1:5000; proteintech,66009-1-Ig) (Rafalo-Ulinska et al., 2016). The bands were visualized by an ECL detection kit (Beyotime Biotechnology, China) using AmerSham Imager 600 (GE, United States). β-actin was used as a loading control.

### Biochemical Detection

Index of oxidative injury (lipoperoxidative damage) was determined by malondialdehyde (MDA) and antioxidant capacity was estimated by superoxide dismutase (SOD). After treatment, the cells were collected to detect MDA and SOD levels using a MDA (A003-2) or SOD (A001-1) kit (Nanjing Jiancheng Biotechnology Research Institute, Jiangsu, China) according to the manufacturer recommendations (Dai et al., 2018; Yan et al., 2019).

### Mitochondrial Reactive Oxygen Species Detection

The intramitochondrial production of ROS in live HT22 cells was detected using the fluorescent MitoSox probe (M36008;Invitrogen) (Roelofs et al., 2015; Chen et al., 2019). The cells were reacted with a 5 μM working solution of MitoSOX Red for 10 min at 37°C, and then carefully washed twice with HBSS. Then, cells were stained with the Hoechst 33342 (1 mg/ml, Beyotime Biotechnology, China) dye for 10 min at 37°C and washed twice with HBSS. Images were obtained with a Laser Scanning Confocal Microscope (OLYMPUS, Japan), and the fluorescence intensity was evaluated with the Image J software (National Institutes of Health, MD, United States). The data are shown as the mean intensities.

### Detection of Mitochondrial Membrane Potential (MMP, Δψm) Using JC-1

MMP was estimated by flow cytometry after staining with JC-1 fluorescent dye (Beyotime Biotechnology, China). MMP is high and JC-1 predominantly appears as red fluorescence when the cell is in a normal state. When the cell is in an apoptotic or necrotic state, the MMP is reduced and JC-1 appears as a monomer indicated by green fluorescence. Approximately 1 × 10^5^ cells in 6-well plates were treated separately. The cells were then washed with PBS and incubated with JC-1 working solution for 20 min at 37°C in the dark. After the incubation, the supernatant was discarded after centrifugation at 600 g/min at 4°C for 3 min and resuspended in 500 μl JC-1 staining buffer. The stained cells were analyzed by flow cytometry to determine the change in the florescence from red to green (Jin et al., 2018).

### Detection of Intracellular Zinc Ions Concentration

Zinquin ethyl ester is thought to be TSQ analog and can form a fluorescent complex with zinc ions to detect intracellular free zinc (Grabrucker et al., 2014). The cells were cultured in confocal microplates and treated separately 500 μl of 2.4 μM Zinquin ethyl ester solution (diluted in DMEM, Dojindo Molecular Technologies, Kumamoto, Japan) was added to the cells and incubated for 60 min at 37°C and washed twice with HBSS. Images were obtained with a Laser Scanning Confocal Microscope, and the fluorescence intensity was evaluated with the Image J software (National Institutes of Health, MD, United States). The data are shown as the mean intensities.

### Statistical Analysis

All data in this study were statistically analyzed using SPSS 23.0 software. The measurement data is represented by the mean ± standard deviation, and the normality of the Shapiro-Wilk test is used. T test or analysis of variance was used for comparison between groups for normal distribution; the rank sum test was used for comparison between groups for abnormal distribution. The chi-square test was used to compare the count data between groups. *P < 0.05, **P < 0.01 indicates that the difference is statistically significant. The data are all obtained by more than three independent experiments. All statistical images are produced using GraphPad Prism version 8.0 software.

## Results

### Determination of Infarct Volume

**FIGURE 2:**
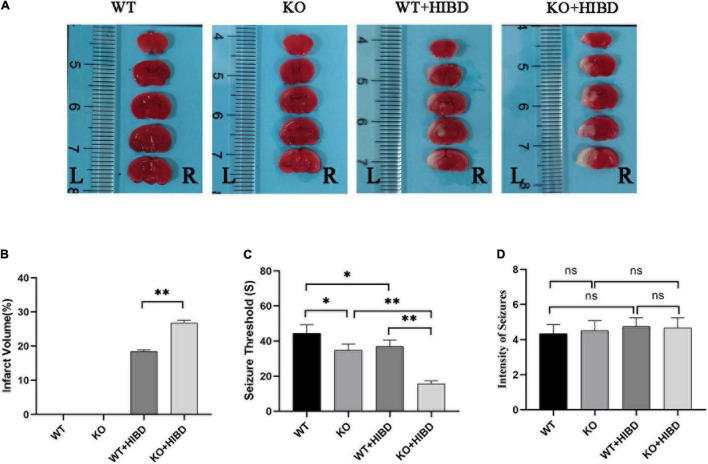
TTC staining, penicillin-induced seizure threshold and intensity of seizure**. (A)** Results of TTC staining. **(B)** Results of infarct volume (*t* test, *n* = 3). **(C)** Seizure threshold (*t*-test, *n* = 6). **(D)** Intensity of seizure (Kruskal-Wallis, *n* = 6). Error bars represent mean ± SD. Ns, not significantly; *P < 0.05; **P < 0.01.

Both WT and *Plppr5*^–/–^ mice showed obvious infarct on the left side of the brain 24 h after HIBD, and the infarct volume of *Plppr5*^–/–^mice was larger than WT mice (Figures 2A,B).

### Determination of Seizure Threshold

Results show that mice in each group had seizures successively. No mice died in the WT group, two mice died in KO group and WT + HIBD group, respectively (mortality rate of 33.3%), three mice died in KO + HIBD group (mortality rate of 50%). Fisher exact test (2 × C) showed that there was no significant difference in mortality among the four groups (*P* = 0.392). The seizure threshold of *Plppr5*^–/–^ mice was significantly lower than that of WT mice (*P* = 0.0102), which shows that *Plppr5* knockout decreased the seizure threshold induced by penicillin in mice. After HIBD treatment, both WT mice and *Plppr5*^–/–^ mice had a lower seizure threshold than the corresponding sham group (*P* < 0.01). Hypoxic-ischemic injury can also reduce the seizure threshold and *Plppr5*^–/–^ mice were more likely to have convulsion (*P* < 0.01), *Plppr5* knockout can aggravate the lower degree of convulsion threshold induced by HIBD. However, there was no significant difference in the intensity of seizures among all groups (*P* > 0.05) (Figures 2C,D).

### Timm Staining

**FIGURE 3:**
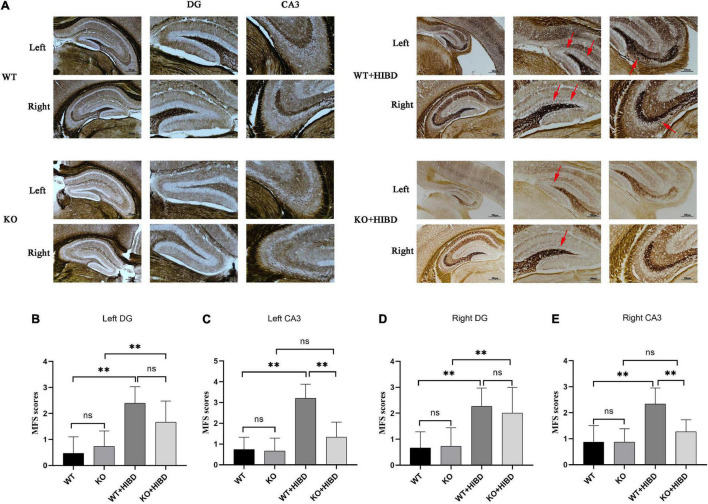
Timm staining. **(A)** Timm staining results. **(B)** MFS scores in DG region of the left hippocampus. **(C)** MFS score in CA3 region of the left hippocampus. **(D)** MFS score in DG region of the right hippocampus. **(E)** MFS score in CA3 region of the right hippocampus. The arrows show where the moss fibers sprouting. Error bars represent mean ± SD. Ns, not significantly; *P < 0.05; **P < 0.01. (Kruskal-Wallis with Bonferroni *post hoc* tests, *n* = 3).

As can be seen from the results of Timm staining, for WT mice, four weeks after HIBD, the mossy fiber sprouting (MFS) scores in bilateral hippocampus CA3 and DG were higher than the sham group (*P* < 0.05). For Plppr5^–/–^ mice, four weeks after HIBD, the MFS scores in bilateral hippocampus DG were higher than the sham group (*P* < 0.05). Furthermore, the MFS scores in bilateral hippocampus CA3 were higher in KO + HIBD group than in WT + HIBD group (KO + HIBD vs. WT + HIBD, *P* < 0.05) (Figure 3).

### Western Blot

**FIGURE 4:**
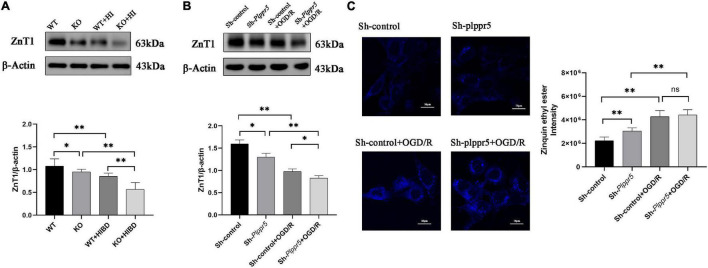
Expression of ZnT1 in mice and HT22 cells and intracellular zinc ions concentration in HT22 cells. **(A)** Expression of ZnT1 in mice of each group; **(B)** Expression of ZnT1 in HT22 cells of each group; **(C)** Intracellular zinc ions concentration. Error bars represent mean ± SD, ns, not significantly; *P < 0.05; **P < 0.01 (*t*-test, *n* = 3).

Western blot results showed that the expression level of ZnT1 was significantly decreased in Plppr5^–/–^ mice with sham treatment than WT mice (*P* < 0.05). And the expression level of ZnT1 in WT mice and Plppr5^–/–^ mice four weeks after HIBD treatment were significantly higher than those in mice treated with sham operation (*P* < 0.05). Four weeks after HIBD treatment, Plppr5^–/–^ mice had higher expression of ZnT1 than WT mice (KO + HIBD vs. WT + HIBD, *P* < 0.05) (Figure 4A and Supplementary Figures 1S1,S2). We found the same results at the cellular level (Figure 4B and Supplementary Figures 1S3,S4).

### Measurement of Intracellular Zinc Ions Concentration

In Sh-*Plppr5* group, the intracellular free zinc content was significantly higher than those in Sh-control. OGD/R treatment can increase the intracellular free zinc in both groups. However, the content of intracellular free zinc in Sh-*Plppr5* + OGD/R group was higher than that in Sh-control + OGD/R group, but the difference was not statistically significant (Figure 4C).

### Mitochondrial Reactive Oxygen Species Detection

**FIGURE 5:**
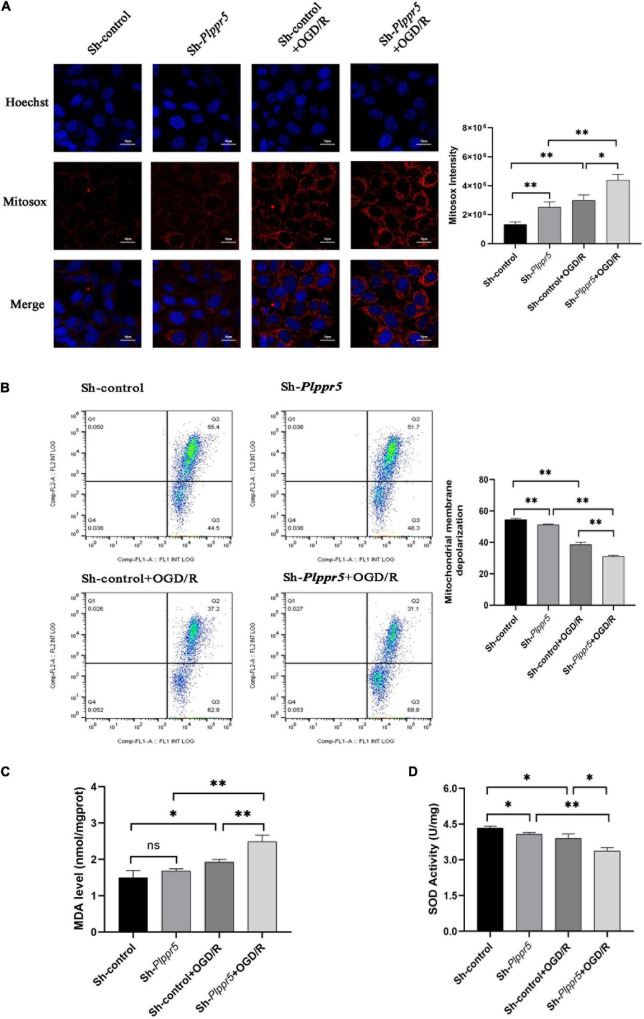
MDA, SOD and ROS MMP levels measurement. **(A)** Mitochondrial ROS content determination and statistics (Red fluorescence: Mitosox; Blue fluorescence Hoechst); **(B)** MMP levels; **(C)** MDA level; **(D)** SOD activity. Error bars represent mean ± SD; ns, not significantly; *P < 0.05; **P < 0.01 (*t*-test, *n* = 3).

*Plppr5* knock out can significantly increase the content of ROS in HT22 cells (Sh-*Plppr5* vs. Sh-control, *P* < 0.01). Meanwhile, after OGD/R treatment, the content of ROS was significantly higher than that in the respective control group in Sh-*Plppr5* and Sh-control group (*P* < 0.01). What’s more, after OGD/R treatment, the content in Sh-*Plppr5* group increased more significantly than that in Sh-control group (Figure 5A).

### Detection of Mitochondrial Membrane Potential

The experimental results showed that the mitochondrial membrane potential of Sh-*Plppr5* group was significantly higher than that of Sh-control group. After OGD/R treatment, the mitochondrial membrane potential was significantly lower than that in the respective control group in Sh-*Plppr5* and Sh-control group. Furthermore, the decrease degree of Sh-*Plppr5* group after OGD/R was more obvious than that of Sh-control group (Figure 5B).

### Biochemical Detection

Results showed that there was no statistical difference in MDA content between Sh-control group and Sh-*Plppr5* group (*P* = 0.188). However, OGD/R treatment can significantly increase the MDA content in both groups, especially in Sh-*Plppr5* group (Figure 5C, sh-control vs. sh-control + OGD/R, *P* = 0.023; sh-*Plppr5* vs. sh-*Plppr5* + OGD/R, *P* = 0.0017).

Knockdown of *Plppr5* decreased the activity of SOD. Compared with Sh-control group, SOD activity in Sh-*Plppr5* group was significantly decreased (*P* = 0.0154). Meanwhile, the SOD activity were decreased after OGD/R treatment in both groups and the decrease was more obvious in the Sh-*Plppr5* group (Figure 5D, sh-control vs. sh-control + OGD/R, *P* = 0.022; sh-*Plppr5* vs. sh-*Plppr5* + OGD/R, *P* = 0.0013).

## Discussion

In this study, we explored the long-term effects of *Plppr5* knockout on the seizure threshold, hippocampal MF regenerative sprouting, and ZnT1 protein expression after HIBD. In addition, by using an *in vitro* oxygen glucose deprivation reperfusion (OGD/R) HT22 cell model, we further analyzed the effects of knocking down *Plppr5* on mitochondrial function, mitochondrial oxidative stress levels, intracellular free zinc content and ZnT1 protein expression. We found that *Plppr5* knockout aggravated hypoxic-ischemic brain damage in mice. This damage effect may be mediated by hippocampal mitochondrial oxidative stress and zinc homeostasis signals. In summary, our data shows a previously unexplored mechanism that *Plppr5* exerts its neuroprotective effect after HIBD by maintaining zinc signal-mediated mitochondrial homeostasis.

Epilepsy is one of the common long-term sequelae of HIBD (Tekgul et al., 2006; Rafało-Ulińska et al., 2020). Basic research has also proved that HIBD in the neonatal period can induce long-term brain spontaneous EEG seizures (Hu et al., 2017). It was found that whether or not they have experienced neonatal hypoxic-ischemic injury, knocking out *Plppr5* can shorten the seizure latency. Specifically, the seizure latency of the two knockout groups was significantly lower than the corresponding control group (KO vs. WT; KO + HIBD vs. WT + HIBD). Since the decrease in the latency of seizure represents an increase in brain excitability, this result indicates that knocking out *Plppr5* can change the excitability of the brain, making animals more prone to seizures. Therefore, *Plppr5* is essential for maintaining the balance of brain excitability and inhibitory activity. We previously found that the reduction of the seizure threshold is related to the up-regulation of the expression of c-fos, a marker for the level of metabolic activity of hippocampal neurons (Sharp et al., 1993; Ni et al., 2005). Mitochondria are the key organelle for metabolic regulation. It has been demonstrated that phosphatidic acid can directly regulate the homeostasis of mitochondrial membrane potential. Pretreatment with autophagy inhibitor cyclosporin A can prevent phosphatidic acid-induced decline in mitochondrial membrane potential and neuronal apoptosis (Holtsberg et al., 1998). This may explain why PRG-1 regulates synaptic excitatory transmission through lipid phosphate-mediated signal transduction (Trimbuch et al., 2009). Therefore, it is reasonable to speculate that *Plppr5*, as a new type of phosphatidic acid phosphatase, may regulate the seizure threshold by regulating mitochondrial homeostasis of hippocampal neurons, which merit further investigation.

In order to further reveal the underlying molecular mechanism, we subsequently examined the expression of ZnT1 protein in the hippocampus by Western blot analysis. Interestingly, compared with the two corresponding sham operation control groups (WT, KO), the two hypoxic-ischemic injury groups (WT + HIBD, KO + HIBD) significantly reduced ZnT1 protein levels, while *Plppr5* gene knockout further down-regulated ZnT1 expression (KO + HIBD and WT + HIBD). It is worth noting that the expression of ZnT1 is also statistically different between the WT and KO groups, which mean that the expression trend of ZnT1 is completely consistent with the changes in seizure threshold. Therefore, it is reasonable to speculate that ZnT1 is related to the reduction of seizure latency caused by *Plppr5* gene knockout. In addition to the role of ZnT1 in transporting zinc from the intracellular to the extracellular space, ZnT1 is also critical to the integrity of the postsynaptic density (PSD) (Doboszewska et al., 2019). ZnT1 directly binds to the cytoplasmic tail of the GluN2A subunit of NMDAR at the PSD of hippocampal neurons and forms a new NMDAR binding protein (Popescu, 2015). Overexpression of zinc-sensitive ProSAP1/Shank2 or ProSAP2/Shank3 can lead to an increase in synaptic density, while the depletion of synaptic zinc, along with the knockdown of zinc-insensitive Shank1, which is insensitive to zinc, lead to the rapid disintegration of PSD and the loss of several postsynaptic molecules including the N-methyl-D-aspartate receptor (NMDAR) (Grabrucker et al., 2011). Therefore, it is reasonable to speculate that ZnT1 plays a more critical role in the long-term seizure threshold reduction caused by neonatal hypoxic-ischemic injury by regulating postsynaptic protein, which merit further investigation.

Interestingly, treatment of hippocampal HT22 neuron cultures silenced by *Plppr5*, with or without OGD/R treatment, markedly decreased the expression of mitochondrial ZnT1 protein. Furthermore, the silencing of *Plppr5* gene directly increased intracellular zinc ion levels, and reduced the mitochondrial membrane potential. It has been shown that mitochondrial dysfunction-induced high-level release of labile Zn^2+^ from mitochondrial aggravates oxidative stress injury (Horn et al., 2013; Turan and Tuncay, 2021). Meanwhile, loss of enzymes involved in mitochondrial phospholipid biosynthesis can affect the assembly and stability of the mitochondrial protein import machinery and cause abnormal mitochondrial morphology or even lethality (Horvath and Daum, 2013). Combined with the notion that the anti-epileptic effect of ketone bodies is achieved through attenuating the production of mitochondrial reactive oxygen species (ROS) (Kim et al., 2015), thus, our finding, that *Plppr5* silencing leads to increased intracellular zinc ion content, and aggravates the damage of mitochondrial function, support the hypothesis that *Plppr5* knockout precipitates late-onset hypersusceptibility to penicillin-induced juvenile seizures by exacerbating hippocampal zinc signaling-mediated mitochondrial damage.

Here, we also analyzed the role of *Plppr5* in the abnormal sprouting of hippocampal mossy fibers in *Plppr5*^–/–^ mice, and found that *Plppr5* gene knockout significantly reduced the MFS scores in the hippocampal CA3 area of wild-type mice after hypoxia-ischemia (KO + HIBD vs. WT + HIBD). In addition, compared with the WT + HIBD group, the MFS scores of the hippocampal dentate gyrus of the KO + HIBD group also decreased to a certain extent, suggesting that *Plppr5* gene knockout has a certain inhibitory effect on hippocampal granule cell axon regeneration. This is consistent with the study of Broggini et al. (2010). They found that *Plppr5* promoted the formation of neurites and filopodia of primary neurons, while *Plppr5* silencing weakened the formation and growth of neurites.

It should be pointed out that in this study we found that although *Plppr5*^–/–^ mice had significantly lower MFS scores in hippocampal CA3 area after HIBD than WT mice, their seizure threshold was also lower than WT mice (KO + HIBD vs WT + HIBD). Sham-operated WT mice and *Plppr5*^–/–^ mice also have the same phenomenon. This connection between hippocampal MFS and seizure threshold in *Plppr5*^–/–^ mice is consistent with the recent view that MFS is an active phenomenon, possibly a normal adaptive mechanism that is reversible, which might be related to replacement or restoration of lost synaptic contacts, rather than to the formation of recurrent excitatory circuits in dentate granule cells (Buckmaster, 2014; Bittencourt et al., 2015; Yamawaki et al., 2015; Ni et al., 2019).

Taken together, our findings demonstrate for the first time that *Plppr5*-deficient mouse pups exposed to neuronal hypoxia and ischemia showed aggravated acute brain injury and long-term brain excitability compared with the same treated WT pups, which involves both zinc and mitochondrial-dependent metabolic pathways in the hippocampus. These data support further investigation into novel approaches to target trace element zinc-related mitochondrial homeostasis in neonatal HIE.

## Data Availability Statement

The original contributions presented in the study are included in the article/Supplementary Material, further inquiries can be directed to the corresponding author/s.

## Ethics Statement

The animal study was reviewed and approved by the University of Soochow University Animal Care and Use Committee.

## Author Contributions

HN designed the study and wrote the manuscript. YS was the main operator analyzed the data. M-FJ, LL, YL, and DW were the operators of the experiment and were responsible for the statistical analysis of the data. All authors contributed to the article and approved the submitted version.

## Conflict of Interest

The authors declare that the research was conducted in the absence of any commercial or financial relationships that could be construed as a potential conflict of interest.

## Publisher’s Note

All claims expressed in this article are solely those of the authors and do not necessarily represent those of their affiliated organizations, or those of the publisher, the editors and the reviewers. Any product that may be evaluated in this article, or claim that may be made by its manufacturer, is not guaranteed or endorsed by the publisher.

## Supplementary Material

Supplementary Figure 1 Figures of Western Blot. **(S1)** Western blotting for the level of ZnT1 in mouse. **(S2)** Western blotting for the level of actin in mouse. **(S3)** Western blotting for the level of ZnT1 in HT22 cell. **(S4)** Western blotting for the level of actin in HT22 cell.
