# Supplementary figures and images for: Genetic Inhibition of Plppr5 Aggravates Hypoxic-Ischemie-Induced Cortical Damage and Excitotoxic Phenotype
Source: Front Neurosci. 2022 Mar 24;16:751489. doi: 10.3389/fnins.2022.751489 (PMC8987356; doi:10.3389/fnins.2022.751489)

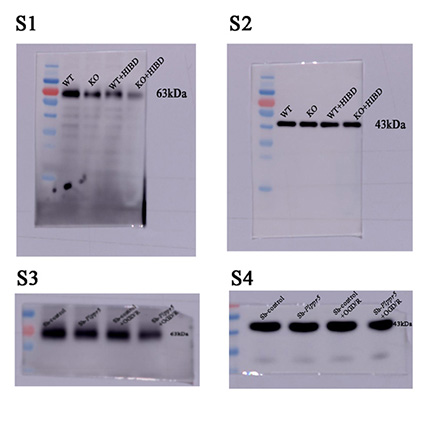

Supplement: Supplementary Figure 1 — Figures of Western Blot. (S1) Western blotting for the level of ZnT1 in mouse. (S2) Western blotting for the level of actin in mouse. (S3) Western blotting for the level of ZnT1 in HT22 cell. (S4) Western blotting for the level of actin in HT22 cell. [file Image_1.JPEG]
